# Structural insights into thyrotropin-releasing hormone receptor activation by an endogenous peptide agonist or its orally administered analogue

**DOI:** 10.1038/s41422-022-00646-6

**Published:** 2022-03-29

**Authors:** Fan Yang, Huanhuan Zhang, Xianyu Meng, Yingge Li, Yingxin Zhou, Shenglong Ling, Demeng Sun, Pei Lv, Lei Liu, Pan Shi, Changlin Tian

**Affiliations:** 1 The First Affiliated Hospital of USTC, School of Life Sciences, Division of Life Sciences and Medicine, Joint Center for Biological Analytical Chemistry, Anhui Engineering Laboratory of Peptide Drug, Anhui Laboratory of Advanced Photonic Science and Technology, University of Science and Technology of China, Hefei, Anhui China; 2 Tsinghua-Peking Joint Center for Life Sciences, Ministry of Education Key Laboratory of Bioorganic Phosphorus Chemistry and Chemical Biology, Department of Chemistry, Tsinghua University, Beijing, China; 3 High Magnetic Field Laboratory, Chinese Academy of Sciences, Hefei, Anhui China

**Keywords:** Electron microscopy, Cell signalling

Dear Editor,

Thyrotropin-releasing hormone (TRH) is a tripeptide (l-pyroglutamyl–l-histidinyl–l-prolinamide) that is widely distributed in the brain and spinal cord, playing dual roles as both an endocrine hormone and a neuropeptide. TRH plays a central role in the hypothalamic–pituitary–thyroid (HPT) axis.^1–3^ TRH is mainly synthesized in the hypothalamus and stimulates the release of thyroid-stimulating hormone (TSH, also known as thyrotropin) and prolactin from the anterior lobe of the hypophysis.^4,5^ TRH activates the class A G protein-coupled receptor (GPCR) thyrotropin-releasing hormone receptor (TRHR), leading to the coupling of Gα_q_/G_11_ and the subsequent production of inositol-1,4,5-triphosphate and the release of intracellular Ca^2+^ ions.^6^ Outside of the hypothalamus, TRH functions as a neurotransmitter or a neuromodulator^2^ and exhibits antidepressant activity, arousal, analeptic and neuroprotective effects, cardiovascular and gastrointestinal autonomic functions.^2,7^ TRH is considered a promising peptide template for the analogue development to treat brain and spinal injuries, or central nervous system (CNS) disorders including epilepsy, schizophrenia, spinal cord trauma, Alzheimer’s disease (AD), Parkinson’s disease (PD) and depression.^7,8^

Despite its high physiological potential, TRH has several shortcomings that have hindered its widely pharmacological application. In particular, TRH has been reported to have a short half-life (< 10 min), highly hydrophilic nature (poor ability to penetrate the blood–brain barrier, BBB), and strong HPT axis-stimulating side effects. Many analogues of TRH have been synthesized in efforts to avoid these undesirable properties. One such analogue, taltirelin (TAL, (1-methyl-(S)-4,5-dihydroorotyl)–l-histidinyl–l-prolinamide), exhibited improved CNS activity and much better pharmacological properties.^8^ TAL has been approved for the treatment of patients with spinocerebellar degeneration (SCD).^7^ TAL is an orally administered TRH analogue, having an 8-time longer effective duration, less affinity, high intrinsic efficacy, and ~100-time more potent CNS stimulant activity than TRH. However, the structural mechanisms underlying the better pharmacological properties of TAL than TRH to TRHR remain unknown.

Herein, our studies started with an evaluation of TRHR activation by TRH or TAL using IP-one accumulation assay. TAL exhibited lower TRHR potency but higher downstream signal transmission efficacy than TRH (Fig. 1a, b), which is consistent with previous functional studies.^9^ To stabilize TRHR–G_q_ complexes, we implemented the NanoBiT tethering strategy to obtain the structures of the TRHR–G_q_ complexes.^10^ The cryo-EM structures of the TRHR–G_q_ complex bound with TRH and TAL were determined at resolutions of 3.19 Å and 3.26 Å, respectively. The electron densities for TRH and TAL in the agonist-binding pockets of the TRHR–G_q_ complex were well defined. This enabled the detailed analysis of the interactions between TRHR and TRH or TAL (Fig. 1c, d; Supplementary information, Figs. S1, S2 and Table S1), providing structural insights into TRHR activation upon agonist binding.

**Fig. 1:**
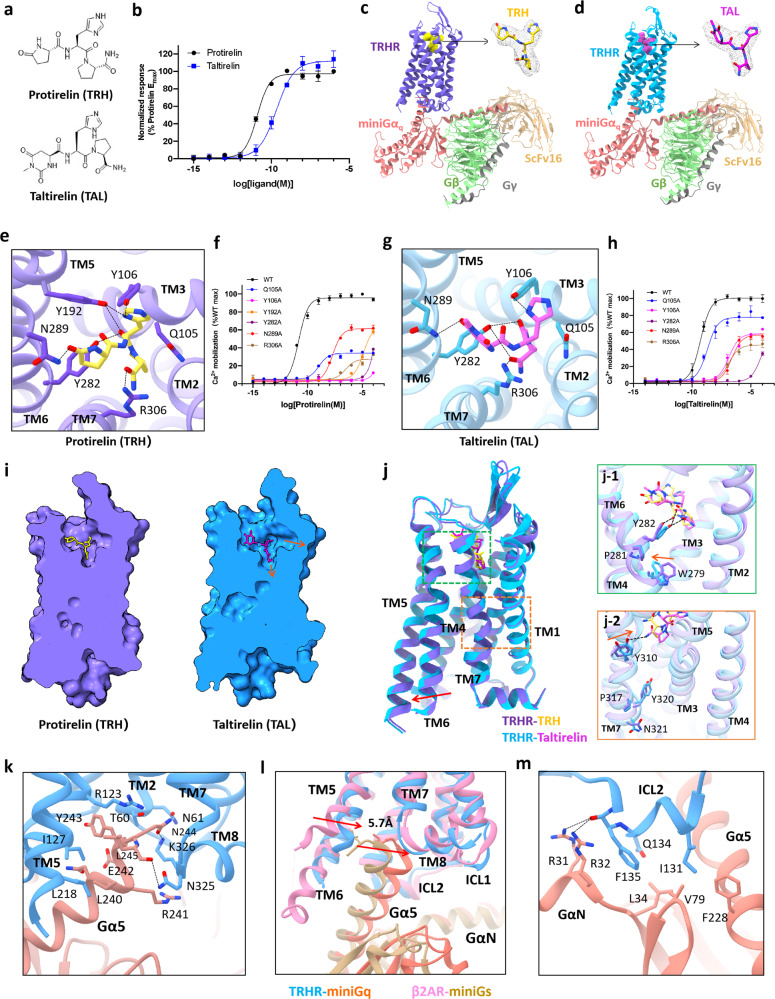
Cryo-EM structures of the human TRHR–G_q_ complex bound with the endogenous peptide TRH or the orally administered peptide analogue TAL. **a** The chemical structures of TRH and TAL. **b** IP-one accumulation assay of TRHR activation by TRH or TAL. Data are expressed as means ± SEM of three independent experiments conducted in triplicate. **c**, **d** Cartoon structural representations of TRH–TRHR–G_q_ (**c**) and TAL–TRHR–G_q_ (**d**) complexes. The ligand model is shown with a surrounding density map. **e** The ligand-binding pocket of TRH is magnified, and the black lines represent electrostatic and hydrogen bond interactions. **f** Ca^2+^ mobilization assay of wild-type and mutant TRHR activation by TRH. Data are expressed as means ± SEM of three independent experiments conducted in triplicate. **g** The ligand-binding pocket of TAL is magnified, and the black lines represent electrostatic and hydrogen bond interactions. **h** Ca^2+^ mobilization assays of wild-type and mutant TRHRs with TAL. Data are expressed as means ± SEM of three independent experiments conducted in triplicate. **i** Space-filling models and cross-sectional views of TRH-bound TRHR and TAL-bound TRHR. **j** Structural comparison of TRH-bound TRHR and TAL-bound TRHR. The rotamer toggle switch and NPxxY motifs are magnified. **k** Network of interactions between TAL-bound TRHR (blue) and the Gα5 helix of Gα_q_ (salmon). **l** Comparison of the Gα5 helix of the TAL–TRHR–G_q_ complex and that of the isoprenaline–β_2_AR–G_s_ complex with the receptors aligned. **m** The TRHR-ICL2–Gα_q_ interface of TAL-bound TRHR (blue) and the Gα5 helix of Gα_q_ (salmon).

The overall structures of the TRH–TRHR–G_q_ and TAL–TRHR–G_q_ complexes have high similarity, with a root mean square deviation (RMSD) value of 0.66 Å for the main chain Cα atoms. However, the interactions between key residues in the agonist-binding pockets of TRHR–G_q_ and TRH or TAL (with chemical modifications on the first pyroglutamyl residue) are distinct, possibly leading to different potency of TRH and TAL at TRHR. In the agonist-binding pocket of the TRH–TRHR–G_q_ complex, Q105^3.32^ and Y106^3.33^ of TM3, Y192^5.39^ of TM5, Y282^6.51^ and N289^6.58^ of TM6, and R306^7.39^ of TM7 form a strong hydrogen bond interaction network to stabilize the binding of TRH (Fig. 1e). Ca^2+^ mobilization assays showed that replacing each of these amino acids with alanine led to remarkably decreased TRH activation compared with wild-type TRHR (Fig. 1f; Supplementary information, Table S2), verifying the critical roles of these amino acids in TRH binding to TRHR, which is consistent with previous biochemistry studies.^11,12^ In the agonist-binding pocket of the TAL–TRHR–G_q_ complex, the distance between N289^6.58^ and the carbonyl at position 6 of the heterocyclohexane of TAL is 3.8 Å, which is longer than the distance between N289^6.58^ and the carbonyl of the lactamized glutamate of TRH (2.6 Å), resulting in a much reduced interaction (Fig. 1g). Moreover, a slight difference in the orientation of the histidine imidazole ring of TAL versus TRH was observed, resulting in a weakened interaction of TAL with Q105^3.32^ and Y106^3.33^ in TM3. The change in histidine orientation in TAL also disrupts the interaction between TAL and Y192^5.39^ (the distance between TAL and Y192^5.39^ is more than 4 Å). Although alanine substitutions of R306^7.39^, Y282^6.51^, N289^6.58^, Q105^3.32^and Y106^3.33^ all significantly impaired the TRHR-activating capacity of TAL, these residues make distinct extents of contributions in TAL-induced TRHR activation versus TRH-induced TRHR activation. In contrast to TRH, substitution of Q105^3.32^ and Y106^3.33^ to alanine resulted in relatively weak potency reduction of TAL in activating TRHR (Fig. 1h; Supplementary information, Table S2).

In addition to the residues in the transmembrane domains of TRHR (TRHR-TMDs), amino acids in the extracellular loop 2 (ECL2) of TRHR also play important roles in stabilizing agonist binding. The ECL2 of TRHR is composed of two β-sheets and is stabilized by the disulfide bond formed between C179 of ECL2 and C98^3.25^of TM3 (Supplementary information, Fig. S4). Both R185^ECL2^ and Y181^ECL2^ were observed to interact with TRH or TAL through hydrogen bonds. These observations are consistent with a previous study reporting that a specific interaction is present between Y181 in ECL2 and the pyroGlu moiety of TRH^12^ (Supplementary information, Fig. S4). Comparison of the space-filling models and cross-sectional views of TRH–TRHR–G_q_ and TAL–TRHR–G_q_ shows that the binding pocket of TAL is wider than that of TRH. Cumulatively, stronger interactions of TRH with the residues in the agonist-binding pocket might explain the higher potency of TRH in activating TRHR, versus TAL (Fig. 1i).

A series of conformational change propagations were observed for TRHR activation upon binding of TRH or TAL. There is a consensus that the expansion of TM6 is a hallmark of GPCR activation and downstream signal transmission. A resting-state structure of TRHR (apo-TRHR, the structure in the absence of any ligands) was retrieved from the library generated using the AI-based program AlphaFold. Relative to the inactive apo-TRHR, the displacement of the cytoplasmic end of TM6 in the TAL-bound and TRH-bound TRHR–G_q_ complexes is 8.4 Å and 8.0 Å, respectively, with respect to Q263^6.32^ (Supplementary information, Fig. S5). In addition, the intracellular end of TM7 moves toward the central cavity by 5.2 Å and 4.3 Å in TAL-bound and TRH-bound TRHR–G_q_, respectively, with respect to L322^7.55^ (Supplementary information, Fig. S5).

Two conserved motifs (the rotamer toggle switch and NPxxY motifs) in cytosolic side of GPCR have been reported to be associated with receptor activation and to be responsible for the displacement of TM6. The stereoisomerism of amidated proline in TRH or TAL leads to obvious conformational changes in these two motifs upon binding of TRH or TAL. First, the phenol side chain of Y282^6.51^ in TRHR was observed to interact with both the backbone carbonyl oxygen and the C-terminal carbonyl oxygen of the third residue (amidated proline) in TAL, while it only interacts with the backbone carbonyl oxygen of the amidated proline in TRH (Fig. 1e, g). The indole side chain of W279^6.48^ in the conserved rotamer toggle switch motif is deflected, making the intracellular end of the TM6 helix of TRHR move further outward upon binding of TAL versus TRH (Fig. 1j-1; Supplementary information, Fig. S6). Second, the hydrogen bond between Y310^7.43^ and the C-terminal-NH_2_ of the amidated proline in TAL results in a conformational change in the conserved NPxxY motif at the cytosolic end of TM7. Additionally, the side chains of Y320^7.53^ and N321^7.54^ exhibit some orientation changes (Fig. 1j**-**2). Theses conformational rearrangements might be related to different efficacies of TAL and TRH in inducing downstream signal transmission.

Structural comparisons between the TRH–TRHR–G_q_ and TAL–TRHR–G_q_ complexes reveal similar interaction interfaces between TRHR and the Gα_q_βγ complex. The Gα5 helix of Gα_q_ is inserted into the central cavity of the receptor and interacts with TM2, TM4, TM5 and TM8 (Fig. 1k). In the structure of TAL-bound TRHR, the T60^2.39^ of the cytosolic end of TM2 can form a hydrogen bond with the N244 of Gα5 (Fig. 1k; Supplementary information; Fig. S7a). The S324^8.47^ and K326^8.49^ at the turning loop of TM7 and TM8 can form hydrogen bonds with N244 of the α5 helix, and N325 can also interact with the carbonyl oxygen of L245 in the α5 helix (Fig. 1k; Supplementary information, Fig. S7b). The close interactions between TM7–TM8 linker of TRHR and Gα_q_ may provide new insights into the binding mode of the G_q_ protein to GPCR.

In GPCR–G_s_ complexes, it is well known that TM5 and TM6 in GPCRs mediate the interaction with Gα5 helix of the Gα_s_ protein, stabilizing the binding of G_s_ protein. In the full agonist isoprenaline-bound β_2_AR–G_s_ complex (PDB: 7DHR), the cytoplasmic side of TM6 moves outward by 14 Å with TM5 extending down two spirals relative to the inactive-state structure (PDB: 2RH1)^13^. For the TAL/TRH-bound TRHR–G_q_ complexes in this study, the intracellular end of TM6 moves outward by only ~8 Å. Moreover, the C-terminus of the Gα5 helix in the TAL–TRHR–G_q_ complex moves 5.7 Å toward the central cavity of the receptor contributing to a close interaction with TM7 and TM8 when compared to the Gα5 helix in the β_2_AR–G_s_ complex (Fig. 1l). In addition, the C-terminus of the Gα5 helix in the TAL–TRHR–G_q_ complex also moves 3.3 Å toward the central cavity when compared with the CCK_A_R–G_s_ complex (PDB: 7EZK), while it is aligned well with the Gα5 of CCK_A_R–G_q_ complex (PDB: 7EZM)^14^ (Supplementary information, Fig. S8). These observations strongly indicate that signal transmission between TRHR and the Gα_q_ protein is accomplished through synergistic interactions mediated by multiple transmembrane helices, which is different from that in other class A GPCR–Gα_s_ complexes.

In addition to the TMDs, the intracellular loops ICL1 and ICL2 of TRHR were also observed to stabilize the interaction with the G_q_ protein. K54^ICL1^ in the TAL–TRHR–G_q_ complex can interact with the carbonyl oxygen in the backbone of H311 and F292 of Gβ, which was not observed in other GPCR–G_q_ structures (Supplementary information, Fig. S7c). In the interface between TRHR-ICL2 and Gα_q_, I131 of ICL2, F228 of the Gα5 helix and V79 of the β2 sheet establish a hydrophobic interaction. F135 of ICL2 and L34 of the β1 sheet are also involved in forming a hydrophobic interaction network (Fig. 1m; Supplementary information, Fig. S7d). Moreover, R31 and R32 of the αN helix can also form hydrogen bonds with the carbonyl oxygen of F135^ICL2^. Comparison of the structures of the TRHR–G_q_ and 5HT_2A_–G_q_ (PDB: 6WHA) complexes indicates that F135^ICL2^ in TRHR is in the similar location of R185 in 5HT_2A_^15^ (Supplementary information, Fig. S9). When F135^ICL2^ in TRHR was substituted by Arginine, Ca^2+^ release upon TRHR activation was significantly decreased, indicating the important role of F135^ICL2^ in stabilizing G protein binding (Supplementary information, Fig. S9). Alignment of the Gα_q_ protein in the TRH–TRHR–G_q_ or TAL–TRHR–G_q_ complex with that of the 5HT_2A_–G_q_ complex demonstrated that the N-terminus of the GαN helix in the TAL–TRHR–G_q_ structure moves up by 7.6 Å relative to that in the 5HT_2A_–G_q_ structure.^15^ The different interactions in the TRHR-ICL2–Gα_q_ interface lead to conformational changes in the αN helix, indicating that the αN helix also plays an important role in signal transduction.

In summary, the cryo-EM structures of the TRHR–G_q_ complex bound to the endogenous agonist TRH and orally administered peptide analogue TAL were determined, respectively. Comparisons of the structures demonstrated stronger hydrogen bond interactions between the Y192^5.39^, N289^6.58^ with TRH versus attenuated interactions with the diazinane-cyclohexane of TAL. Furthermore, the weakened interaction between TAL and TRHR-ECL2 made the ligand-binding pocket of TAL wider than that of TRH. Moreover, structural comparison of TAL-bound TRHR with TRH-bound TRHR suggested that the deflection of the rotamer toggle switch at TM6 and the side chain reorientations in the NPxxY motif at the cytosolic end of TM7 might be responsible for the higher efficacy of TAL versus TRH. These results provide structural insights into the mechanism underlying the lower potency but higher TRHR–Gα_q_ transmission efficacy of TAL versus the endogenous peptide TRH. In addition, compared with other class A GPCR–G protein complexes, notable differences were observed in the TRHR–Gα_q_ interface. Understanding the correlation between the structures and pharmacological properties of TRHR–G_q_ will benefit further rational drug development targeting TRHR and other GPCRs.

## Supplementary information

Supplementary Information

## Data Availability

The cryo-EM density maps and corresponding atomic coordinates of the TRH-bound and TAL-bound TRHR–G_q_ complexes have been deposited in the Electron Microscopy Data Bank and the Protein Data Bank under the accession codes of EMD-32950, EMD-32949 and 7X1U, 7X1T, respectively. All data analyzed in this study are included in this paper and the Supplementary information.
