# Author Correction: Structural insights into thyrotropin-releasing hormone receptor activation by an endogenous peptide agonist or its orally administered analogue

**DOI:** 10.1038/s41422-022-00667-1

**Published:** 2022-05-09

**Authors:** Fan Yang, Huanhuan Zhang, Xianyu Meng, Yingge Li, Yingxin Zhou, Shenglong Ling, Demeng Sun, Pei Lv, Lei Liu, Pan Shi, Changlin Tian

**Affiliations:** 1 The First Affiliated Hospital of USTC, School of Life Sciences, Division of Life Sciences and Medicine, Joint Center for Biological Analytical Chemistry, Anhui Engineering Laboratory of Peptide Drug, Anhui Laboratory of Advanced Photonic Science and Technology, University of Science and Technology of China, Hefei, Anhui China; 2 Tsinghua-Peking Joint Center for Life Sciences, Ministry of Education Key Laboratory of Bioorganic Phosphorus Chemistry and Chemical Biology, Department of Chemistry, Tsinghua University, Beijing, China; 3 High Magnetic Field Laboratory, Chinese Academy of Sciences, Hefei, Anhui China

**Keywords:** Electron microscopy, Cell signalling

Correction to: *Cell Research* 10.1038/s41422-022-00646-6, published online 29 March 2022

In the initial published version of this article, there is an error in the Supplementary information, Figs. S1a and S2a, respectively. In Supplementary information, Fig. S1a, the ordinate of size-exclusion chromatography profile was incorrectly labeled as UV289/mAU, which should be UV280/mAU. And in Supplementary information, Fig. S2a, the gel image of SDS-PAGE analysis was misused. The correct version is shown below.

**Fig. S1a:**
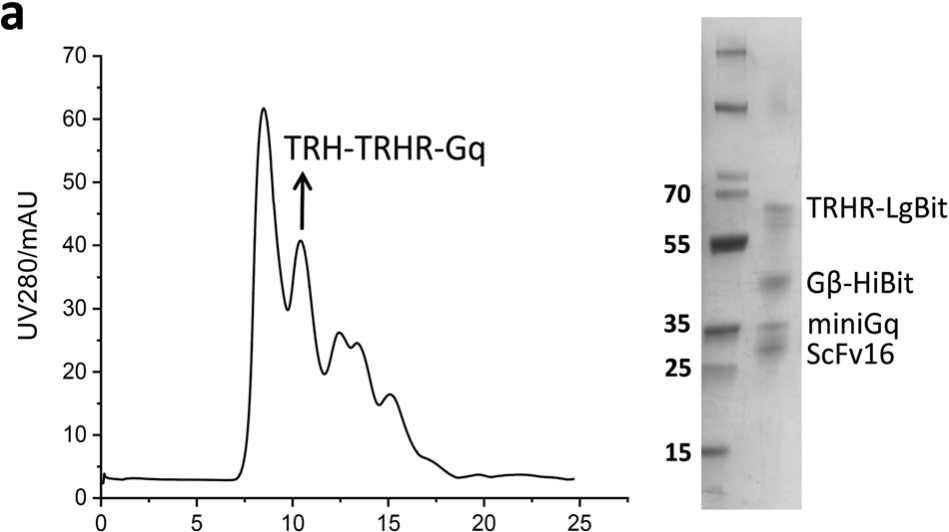
Size-exclusion chromatography profile and SDS-PAGE analysis of TRHR-Gq complex bound with protirelin (TRH).

**Fig. S2a:**
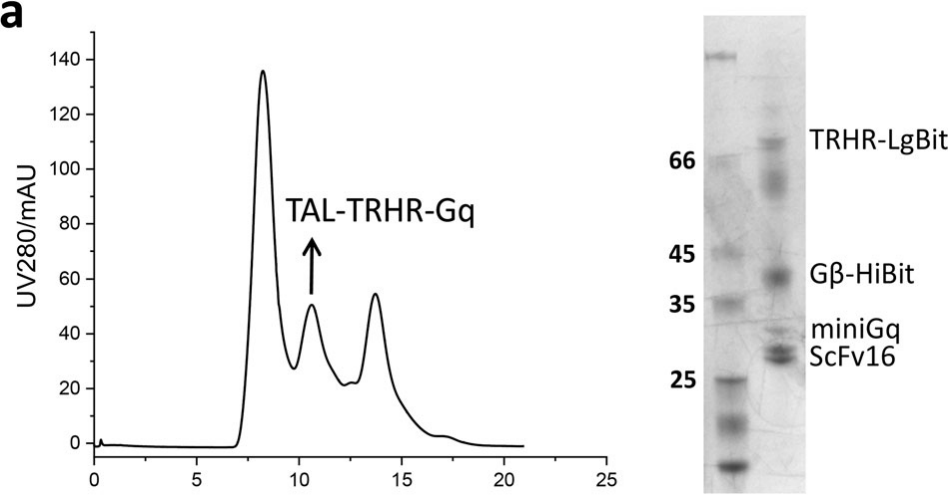
Size-exclusion chromatography profile and SDS-PAGE analysis of TRHR-Gq complex bound with taltirelin (TAL).

These corrections do not affect the major findings and conclusions of this work. We deeply apologize for this oversight.

## Supplementary information

Supplementary information

