# Supplementary material for: Structural insights into thyrotropin-releasing hormone receptor activation by an endogenous peptide agonist or its orally administered analogue
Source: Cell Res. 2022 Mar 29;32(9):858–61. doi: 10.1038/s41422-022-00646-6 (PMC9437008; doi:10.1038/s41422-022-00646-6)
Supplement: Supplementary file 1 — Supplementary Information [file 41422_2022_646_MOESM1_ESM.pdf]

Supplementary Materials for

Structural Insights into Thyrotropin-Releasing Hormone Receptor Activation by an Endogenous Peptide Agonist or its Oral-administered Analogue

Materials and Methods

Construct design

The full-length gene coding sequence of human TRHR was synthesized with no additional mutations or loop deletions. The DNA sequence of TRHR was cloned into *pFastBac1* vector with N-terminal thermostabilized apocytochrome b_{562}RIL (BRIL) epitope followed by a HA signal sequence and FLAG tag, and the C-terminal was attached with a 2 × MBP tag to facilitate the protein expression and purification. The NanoBiT tethering strategy was applied by fusing a LgBiT subunit (Promega) at the receptor C-terminus using homologous recombination to improve protein homogeneity and stability. The mini-Gαq chimeric construct refers to the $5HT_{2A}$-miniGq complex (PDB: 6WHA). All the three G protein complex components, miniGαq, *rat* Gβ1 and *bovine* Gγ2, were constructed into *pFastbac* vector, respectively. The HiBiT (Promega) was fused to the C-terminus of *Rat* Gβ1 with a 15-amino acid (15 aa) polypeptide linker (GSSGGGGSGGGGSSG).

Protein expression and purification

Baculoviruses were prepared using the Bac-to-Bac Baculovirus Expression System (Invitrogen). *Sf9* insect cells were cultured to a density of 3 × 10^6 cells per mL and co-infected with baculovirus encoding TRHR-LgBiT, miniGαq, Gβ1-15AA-HiBiT, Gγ2, and scFv16 protein at the ratio of 1:1:1:1. The transfected sf9 cells were grown at 27 °C for 48 h and then were collected by centrifugation at 1000 x g (Beckman) for 20 min and keep frozen at −80 °C for further usage. The frozen cells were thawed on ice and resuspended in lysis buffer containing 20 mM HEPES, pH 7.5, 100 mM NaCl, 10% (v/v) glycerol, 10 mM $MgCl_2$, 5 mM $CaCl_2$, 100 μM TCEP and supplemented with protease inhibitor cocktail (Apexbt). Cells were lysed by dounce

homogenization and complex formation was initiated with the addition of 10 μg/mL ScFv16, 25 mU/mL Apyrase (Sigma-Aldrich) and 10 μM protirelin or 30 μM taltirelin (MCE) for 2 h at room temperature (RT). The membrane was then solubilized by adding 1.0% (w/v) n-dodecyl-β-d-maltopyranoside (DDM, Anatrace), 0.2% (w/v) cholesteryl hemisuccinate (CHS, Sigma-Aldrich) for 2 h at 4°C. The sample was clarified by centrifugation at 30,000 rpm for 30 min and the supernatant was then incubated with MBP resin (BioLabs) for 3 h at 4℃. After incubation, the resin was loaded into a gravity flow column, followed by wash1 buffer of 20 mM HEPES, pH 7.5, 100 mM NaCl, 5 mM $MgCl_2$, 5 μM protirelin or 10 μM taltirelin, 25 μM TCEP, 0.1% (w/v) LMNG and 0.02% (w/v) CHS and then, with wash 2 buffer of 20 mM HEPES, pH 7.5, 100 mM NaCl, 5 mM $MgCl_2$, 5 μM protirelin or 10 μM taltirelin, 25 μM TCEP, 0.03% (w/v) LMNG and 0.006% (w/v) CHS. Then use 5 column volumes of wash 2 buffer to resuspend the MBP resin and add 0.5 mg TEV enzyme for digestion overnight at 4℃. The purified TRHR-miniGq complex was concentrated using an Amicon Ultra centrifugal filter (molecular weight cut-off of 50 kDa, Millipore) and then loaded on to a Superdex 200 Increase 10/300 GL column (GE Healthcare) that was pre-equilibrated with buffer containing 20 mM HEPES, pH 7.5, 100 mM NaCl, 2 mM $MgCl_2$, 100 μM TCEP, 5 μM protirelin or 10 μM taltirelin, 0.001% (w/v) LMNG and 0.0002% (w/v) CHS. The eluted fractions of monomeric complex were collected and concentrated to 0.8 mg/mL for cryo-EM grids preparation.

Cryo-EM grid preparation and data collection

For the preparation of cryo-EM grids, 3 μL of the purified TRHR-Gq/TRH or TRHR-Gq/TAL complexes at 0.8 mg/ml were applied onto a freshly plasma-cleaned holey carbon grid (GryoMatrix-M024, R1.2/1.3, 300 mesh, Au). Grids were blotted for 9 s and plunge-frozen in liquid ethane cooled by liquid nitrogen using a Vitrobot Mark IV (ThermoFisher Scientific) at 4℃ and with 100% humidity.

Cryo-EM imaging was performed on a Titan Krios electron microscope at 300 kV accelerating voltage using a Gatan K3 Summit direct electron detector with a Gatan energy filter in the Center of Cryo-Electron Microscopy, University of Science and Technology of China (Hefei, China). A total of 3,687 movies for TRHR-Gq/TRH complex and 3,321 movies of TRHR-Gq/TAL complex were collected with a nominal magnification of 81,000 ×, corresponding to a pixel size

of 1.07 Å using the EPU software. Each movies stack was recorded for a total of 3.5 s and 30 frames per micrograph with a defocus range of -1.2 to -2.2 μm.

Image processing and map construction

Dose-fractionated image stacks were subjected to beam-induced motion correction using MotionCor2 (v1.0.6). The processed images were transferred to CryoSPARC (v3.0)[1] and contrast transfer function (CTF) estimation was performed with patch CTF estimation. Following CTF estimation, auto-picking particles were extracted by four-time downscaling resulting in the pixel size of 4.28 Å. After two rounds of 2D classification, the particles from well-defined 2D averages were extracted with a pixel size of 2.14 Å for further ab-initio reconstruction and heterogeneous refinement. After several rounds of heterogeneous refinement, the selected subset of particles from heterogeneous refinement were extracted with a pixel size of 1.07 Å. Followed homogeneous refinement and Non-uniform refinement yielded a density map with 3.19 Å of TRHR-Gq/TRH complex and 3.26 Å of TRHR-Gq/TAL complex.

Model building and refinement

The initial model for the human TRHR receptor was derived from inactive state TRHR which simulated by AlphaFold followed by extensive remodeling using COOT[2]. And the miniGαqβγ heterotrimer was derived from $5HT_2A$-miniGq complex (PDB code: 6WHA). The N-terminal residues 1-24, ICL3 residues 222-261 and C-terminal residues 335-398 of TRHR receptor were not built due to the lack of corresponding densities. Structure refinement and model validation were performed using phenix.real_space_refine module in PHENIX[3]. The final model was subjected to refinement and validation in PHENIX. Figures were prepared using UCSF Chimera[4] or UCSF Chimera X[5].

Ca^{2+} mobilization assay

HEK293T cells were transiently transfected with wide-type or mutant TRHR for 24 h. Then cells were seeded at a density of 6×10^4 cells per well into 96-well culture plates and incubated overnight at 37 °C in 5% CO_2. The Cells were washed with fresh calcium buffer (HBSS (Gibco 14025) supplemented with 20mM HEPES, 0.1% BSA and 2.5mM probenecid; pH 7.4) and then

incubated with 2 μM Fluro-4 AM (Thermo Fisher Scientific, F14201) in calcium buffer at 37 °C for 45 min. The plate was washed once and added with 50 μL calcium buffer per well before measuring. Different concentrations of TRH or TAL were added, followed by an analysis of intracellular calcium mobilization on a FLIPR instrument (Molecular Devices) with excitation at 488 nm and emission at 540 nm. The fluorescence intensity was measured at 1-s intervals for 410 s. Data were normalized to the baseline response of the ligand. Data was analyzed using nonlinear regression in GraphPad Prism software 9.0.

IP-one accumulation signaling assay

HEK293T cells were cultured in DMEM (Gibco, Thermo Fisher Scientific) supplemented with 10% FBS and 1% penicillin/streptomycin at 37°C and 5% CO2. The cells were transiently transfected with wild-type or mutant TRHR using Lipofectamine 3000 (Thermo Fisher Scientific). Twenty-four hours post-transfection, IP1 experiments were performed using an IP-one assay kit (Cisbio, 62IPAPEC) according to the kit instructions. In brief, cells were harvested and seeded in 384-well plates (7 μL, 12000 cells per well) and incubated with increased concentrations of protirelin or taltirelin at 37 °C for 1 h. After incubation, 3 μL of d2-labeled IP1 and 3 μL of cryptate-labeled anti-IP1 antibody (1:20 diluted in lysis and detection buffer) were added to each well. The fluorescence signal was measured by a CLARIOstar microplate reader with excitation at 330 nm and emission at 620 nm and 665 nm. The data were analyzed using GraphPad Prism software 9.0.

Supplementary Reference:

1 Punjani, A. *et al.* cryoSPARC: algorithms for rapid unsupervised cryo-EM structure determination. *Nat. Methods* **14**, 290 – 296 (2017).

2 Emsley, P. & Cowtan, K. Coot: model-building tools for molecular graphics. *Acta crystallographica. Section D, Biological crystallography* **60**, 2126-2132 (2004).

3 Afonine, P. V. *et al.* Real-space refinement in PHENIX for cryo-EM and crystallography. *Acta Crystallogr D Struct Biol* **74**, 531 – 544 (2018).

4 Pettersen, E. F. *et al.* UCSF Chimera--a visualization system for exploratory research and analysis. *J Comput Chem* **25**, 1605-1612 (2004).

5 Pettersen, E. F. *et al.* UCSF ChimeraX: Structure visualization for researchers, educators, and developers. *Protein Sci.* **30**, 70 – 82 (2021).

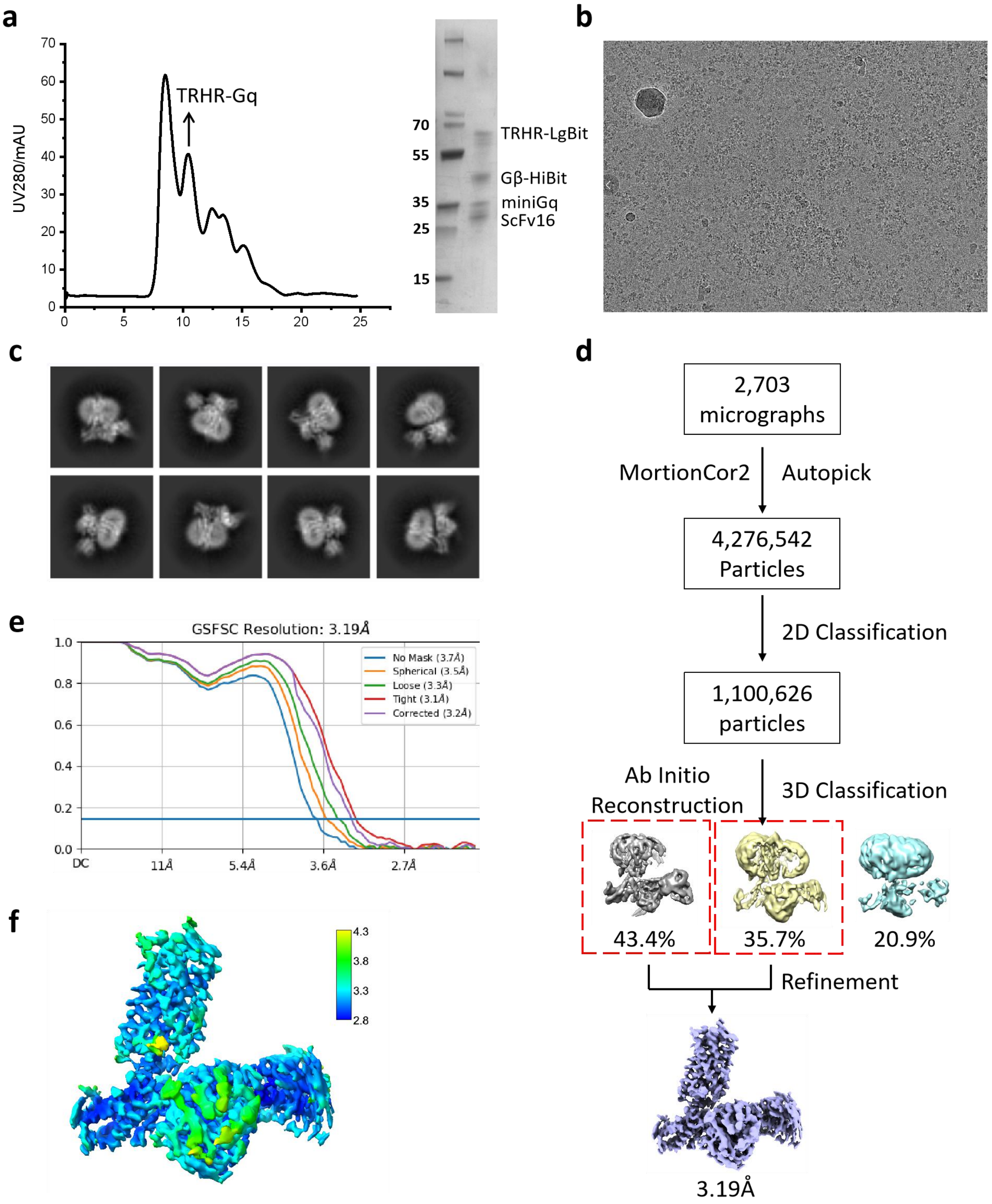

Fig. S1 Purification and cryo-EM data processing of protirelin bound TRHR-Gq complex.

a Size-exclusion chromatography profile and SDS-PAGE analysis of TRHR-Gq complex bound with protirelin (TRH). **b** Representative micrograph after motion correction and dose weighting. **c** 2D class averages of TRHR-Gq complex bound with protirelin (TRH). **d** Flow chart of cryo-EM data processing using cryoSPARC. **e** Gold-standard FSC validation curves from cryoSPARC. f Density map of TRH-TRHR-Gq complex colored by local resolution estimation.

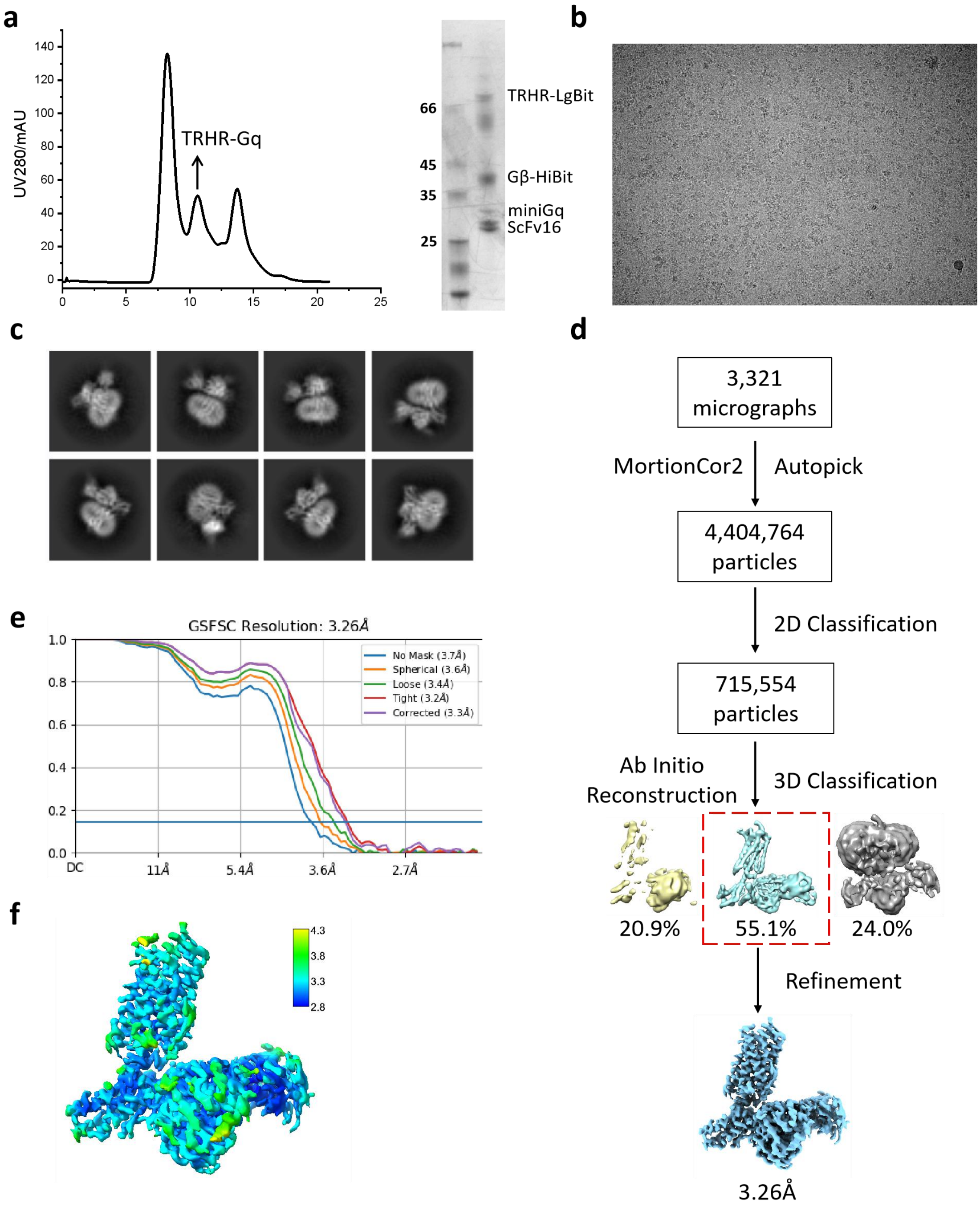

Fig. S2 Purification and cryo-EM data processing of taltirelin bound TRHR-Gq complex.

a Size-exclusion chromatography profile and SDS-PAGE analysis of TRHR-Gq complex bound with taltirelin (TAL). **b** Representative micrograph after motion correction and dose weighting. **c** 2D class averages of TRHR-Gq complex bound with taltirelin (TAL). **d** Flow chart of cryo-EM data processing using cryoSPARC. **e** Gold-standard FSC validation curves from cryoSPARC. f Density map of TAL-TRHR-Gq complex colored by local resolution estimation.

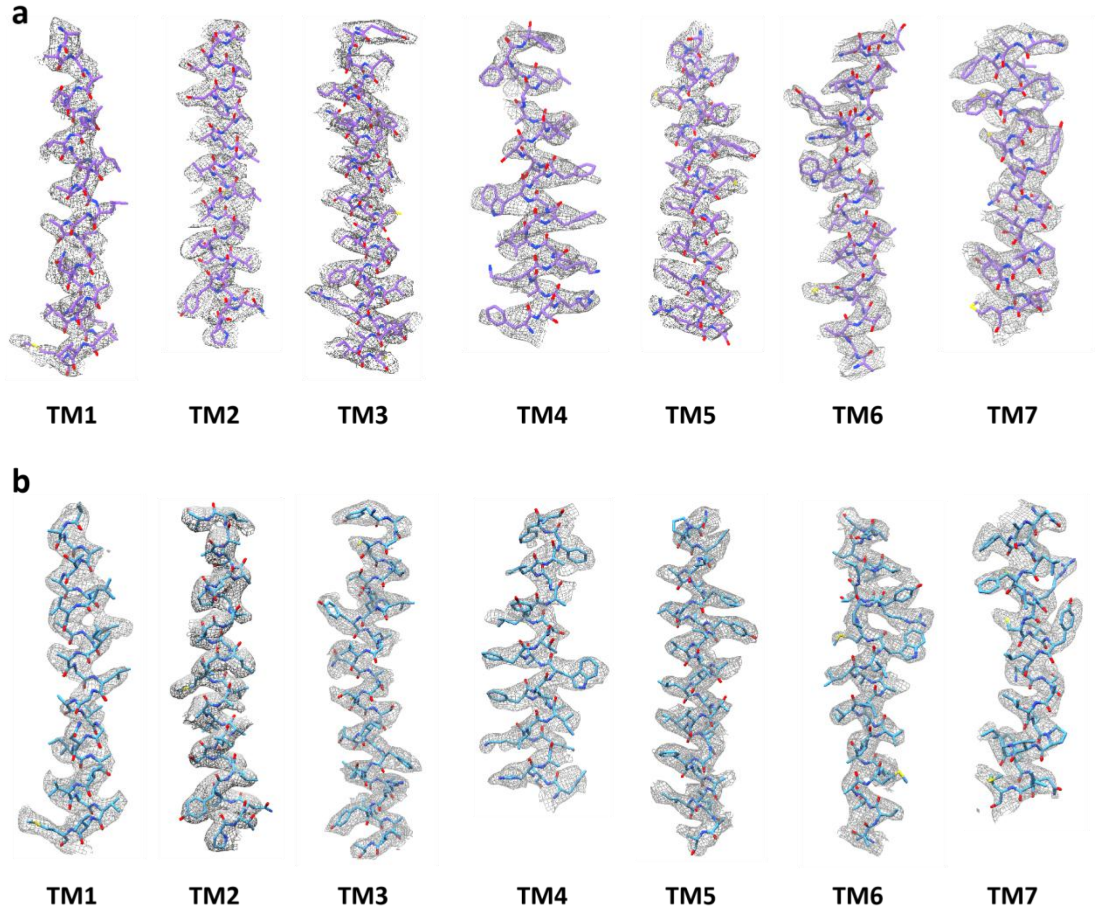

Fig.S3 Cryo-EM densities of representative segments of TRH or TAL bound TRHR-Gq complex.

a,b Cryo-EM density maps and models of transmembrane domain of TRHR-Gq/TRH complex **(a)** and TRHR-Gq/TAL complex **(b)**. The cryo-EM maps are shown in mesh and molecular models as stick representation.

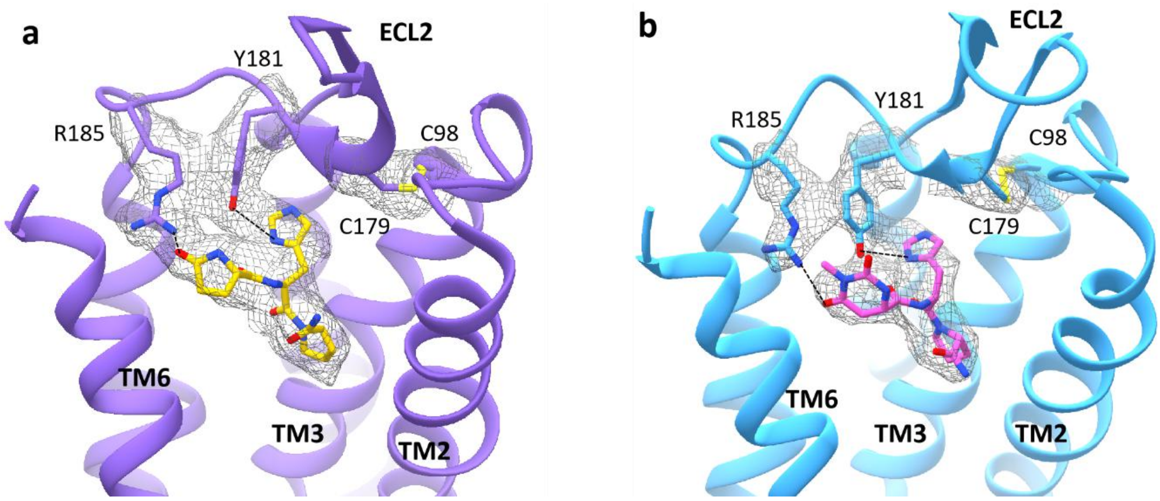

Fig. S4 The ECL2 interactions with TRH and TAL.

a,b ECL2 interactions of TRH (yellow)-bound TRHR (purple)**(a)**. ECL2 interactions of TAL (magenta)-bound TRHR (blue)**(b)**. The density maps of R185, Y181, C179, and both ligands are shown in mesh.

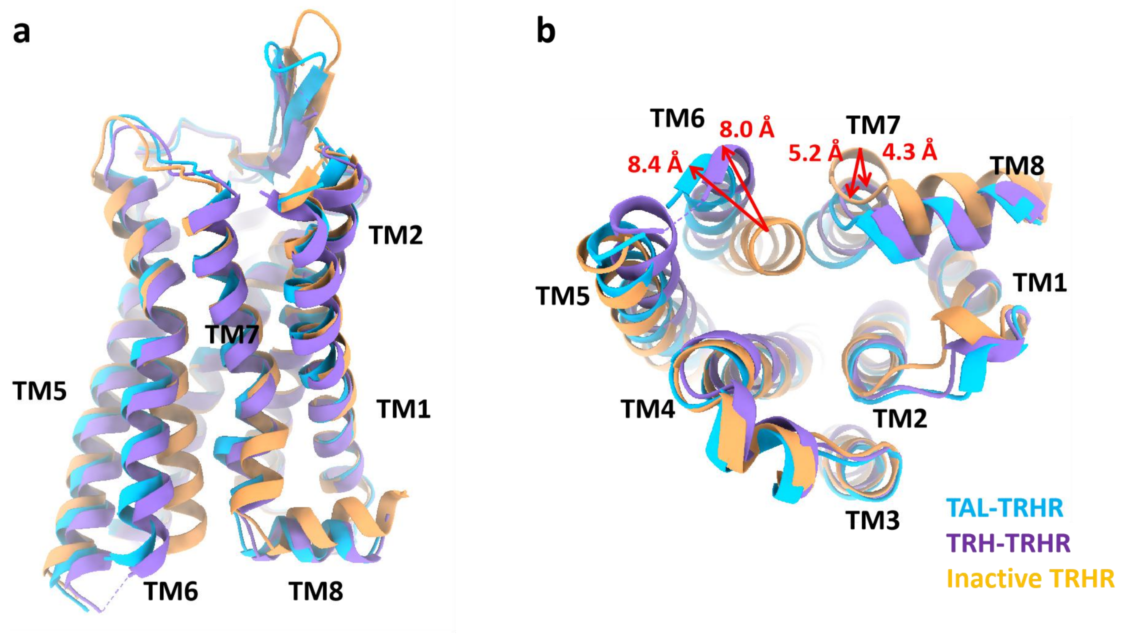

Fig. S5 Conformational changes after activation of TRHR receptor.

a,b Side **(a)** and top **(b)** views of alignment of active TAL-bound TRHR (blue), TRH-bound TRHR (purple) and alphafold simulative inactive (orange) TRHR receptor.

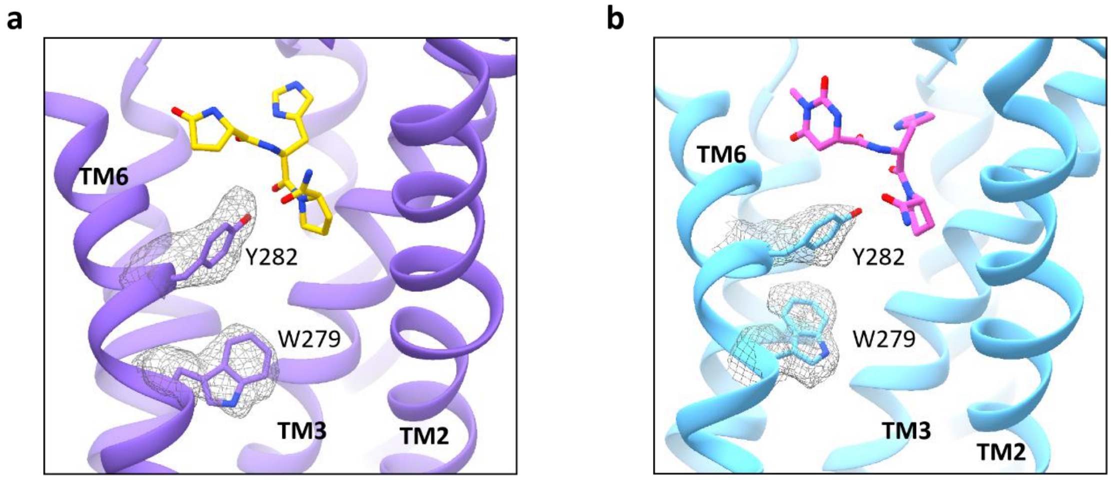

Fig. S6 The electron density maps of "toggle switch" in TRH or TAL -bound TRHR.

The density maps of Y282 and W279 are shown in the TRH**(a)** and TAL**(b)** -bound TRHR.

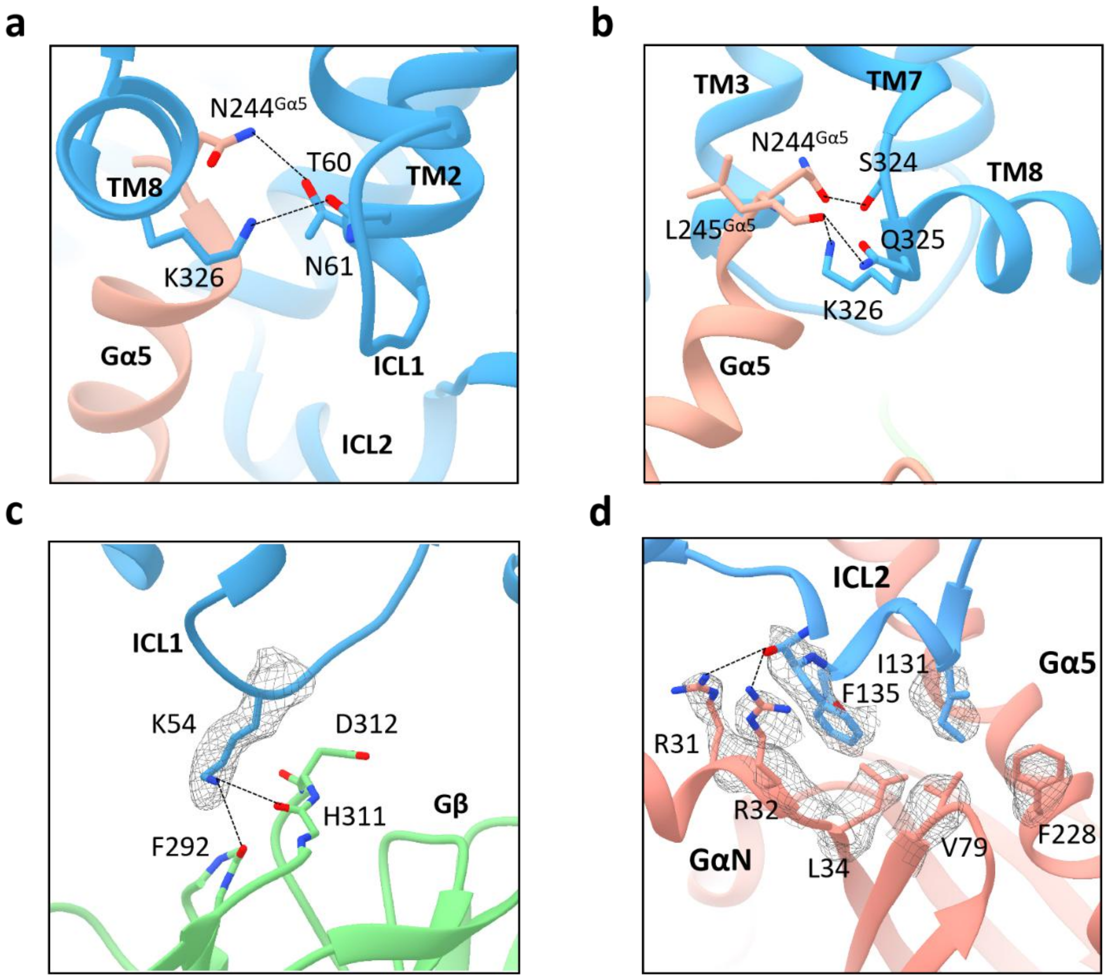

Fig. S7 The interaction interface of TAL-bound TRHR and G protein.

a The TM2 and TM8 work together to stabilize the interaction with Gα5 helix. **b** The interaction interface of TM7-TM8 linker and Gα5 helix of TRHR-Gq/TAL complex. **c,d** The interaction interface between ICL1**(c)** and ICL2**(d)** with G protein of TAL-TRHR-Gq complex. The local density maps are shown in mesh.

a

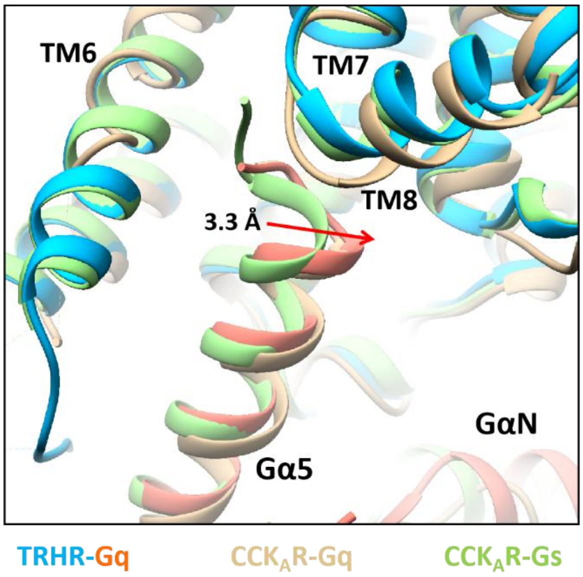

Fig. S8 Comparison of TAL-bound TRHR-Gq, CCK$_A$R-Gq and CCK$_A$R-Gs complexes.

a Compare the Gα5 helix of TAL-bound TRHR-Gq (Salmon) with that of the CCK$_A$R-Gq (wheat)(PDB:7EZM) and CCK$_A$R-Gs complex (green)(PDB:7EZK).

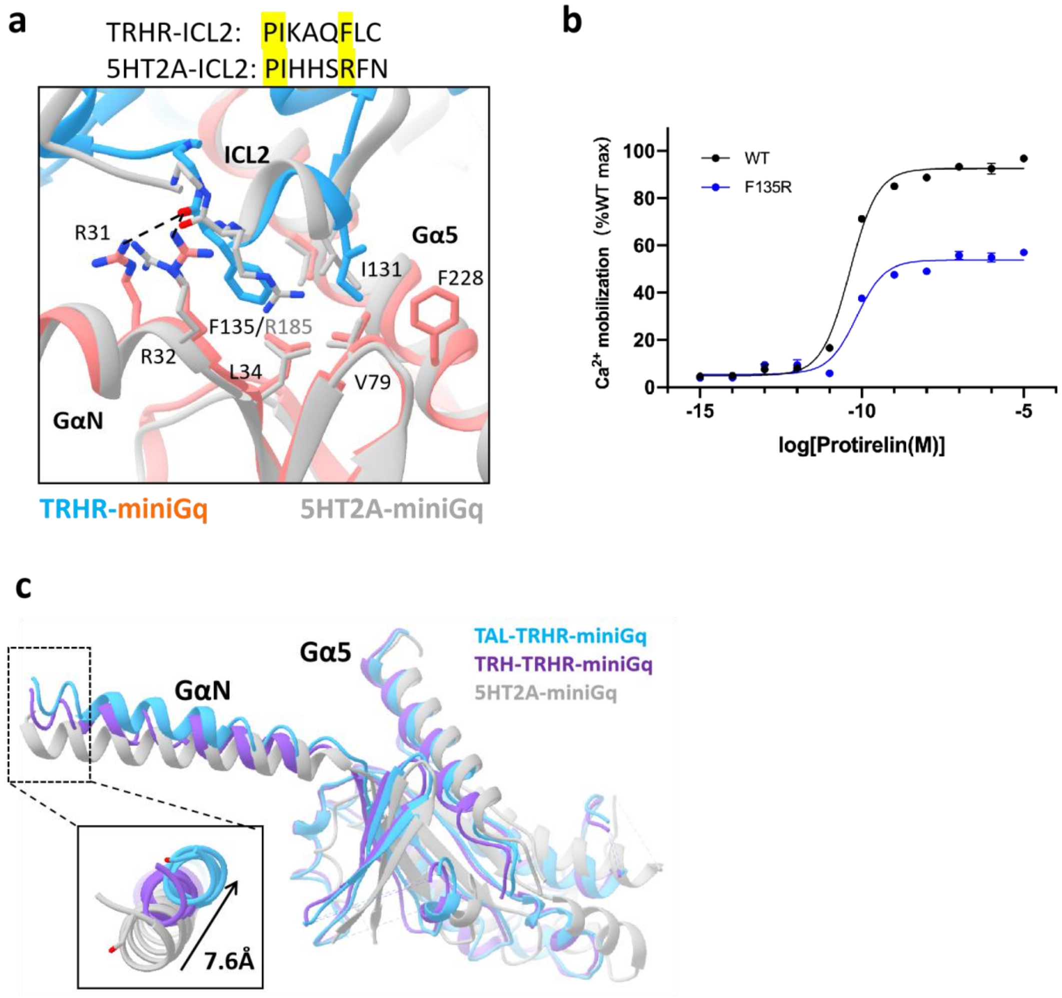

Fig. S9. The role of ICL2 in G protein signal regulation.

a Structure comparison of TAL bound TRHR-Gq complex and $5HT_{2A}$-Gq complex with ICL2-Gq interface. The amino acids that interact with G proteins are shown in yellow. **b** Functional experiments indicate that TRHR may adopt different signaling pathway selection patterns from $5HT_{2A}$. The data represent mean ± s.e.m. of three independent experiments performed in triplicates. **c** Structure comparison of the Gq proteins of TAL-TRHR, TRH-TRHR and $5HT_{2A}$-miniGq complex.

Table S1 | Statistics of cryo-EM data collection, 3D reconstruction and model refinement.

**Data Collection**		
**Protein**	TRH-TRHR	TAL-TRHR
**Microscope**	FEI Titan Krios	FEI Titan Krios
**Voltage (kV)**	300	300
**Detector**	Gatan K3 Summit	Gatan K3 Summit
**Detector mode**	Super	Super
**Pixel size (Å)**	0.535	0.535
**Defocus range (μm)**	-1.2 ~ -2.2	-1.2 ~ -2.2
**Electron dose (e^-/Å^2)**	55	55
**Frames per image**	30	30
**Exposure time (s)**	3.5	3.5
**Number of images**	2,703	3,321
**3D reconstruction**		
**Final Particle number**	870,595	394,270
**Symmetry**	C1	C1
**Overall resolution (Å)**	3.19	3.26
**Model refinement**		
**Model composition**		
**Chains**	6	6
**Ligands**	LIG:1	LIG:1
**Non-hydrogen atoms**	7,494	7,482
**Protein residues**	1,079	1,078
**Bonds (RMSD)**		
**Length (Å)**	0.006	0.005
**Angles (°)**	1.010	0.940

**Ramachandran plot (%)**		
**Outliers**	0.00	0.00
**Allowed**	9.74	8.65
**Favored**	90.26	91.35
**Rotamer outliers (%)**	0.76	1.20
**MolProbity score**	1.87	1.84
**Clash score**	6.02	5.10

Table S2 | Ca^{2+} mobilization assay of wild-type and mutant TRHRs activation by Protirelin or Taltirelin. pEC_{50} values represent the negative logarithm of agonist concentration that produces half maximal response. Emax values are maximal response as percentage of wild-type TRHR response. All values are expressed as mean ± s.e.m. of three independent experiments conducted in triplicate.

	**Protirelin**				**Taltirelin**				**Expression** (% of WT)
	EC50 (nM)	Fold shift	Mean±SEM(n)		EC50 (nM)	Fold shift	Mean±SEM(n)		
			pEC_{50}	Emax			pEC_{50}	Emax	
WT	0.014	1	10.85±0.03(3)	100(3)	0.453	1	9.37±0.04(3)	100(3)	100(6)
Q105A	1.833	129	8.77±0.12(3)	42.84±5.16(3)	3.637	8	8.16±0.18(3)	73.48±2.24(3)	76.36±3.91(3)
Y106A	nd	nd	nd	nd	264.6	585	6.30±0.18(3)	51.21±4.87(3)	98.82±4.73(3)
Y192A	8163	574454	5.13±0.13(3)	62.16±4.88(3)	/	/	/	/	120.67±8.77(3)
Y282A	nd	nd	nd	nd	nd	nd	nd	nd	83.36±3.52(3)
N289A	30.01	2112	7.57±0.14(3)	66.16±3.85(3)	322.2	712	6.51±0.07(3)	53.60±3.00(3)	60.84±3.36(3)
R306A	282.30	19904	6.55±0.02(3)	39.71±6.43(3)	608.5	1345	6.12±0.27(3)	44.97±7.32(3)	52.34±0.83(3)
F135R	0.028	2.0	10.60±0.14(3)	55.21±5.13(3)					119.70±1.46(3)
